# Medical Properties of Ox Gall

**Published:** 1853-09

**Authors:** 


*Medical Properties of Ox Gall.*—We then propose to treat those affections arising from deficiency either in quality or quantity of the bilious secretion, by administering that which will prove, as we think it will in many instances, a substitute for it. If we may believe the testimony of several distinguished medical philosophers and practitioners who have used the ox bile in dyspepsia, and in its almost infinite variety of attendants, we will be fully convinced of its great utility. It generally corrects the acidity of the stomach and consequent headache which so often harass dyspeptic persons. Its alkaline properties seem to counteract the acid, and thus remove all the symptoms depending upon it. Cases are mentioned, upon the highest authority, in which the patient, after resorting to every article in the list of cathartics, in order to remove the constipated habit so frequently attending dyspepsia, have received permanent relief from the use of the inspissated ox gall. It has been used in cases in which the blue pill was inadequate to the purpose of producing any but the most transient effect, and in which all purgatives would leave the bowels in the same, if not a worse, condition than that in which they found them. The patients were compelled to linger on for three or four years, being almost daily under the painful necessity of taking cathartics, which would produce much griping and general uneasiness after taking them. Under these perplexing circumstances, the article to which we have called attention, has been administered in the form of a pill with the happiest effect. One of these pills—5 grs.—has been given every three hours, and at these rates they scarcely ever fail to produce, in less than twenty-four hours, full stools of natural consistence, and that, too, without any pain whatever attending their operation. Hfter taking one of these pills twice or thrice per day for five or six days, the acidity almost always leaves the stomach, also the headache subsides, and the bowels resume their natural and healthy condition.

The first instance in which I have used the inspissated ox bile, was in the case of Mr. R., aged 25 years, an intelligent young gentleman of my acquaintance, who had been laboring under dyspepsia, attended with slight hypochondria and the most obstinate constipation. He was of a delicate, nervous temperament, and much given to sedentary habits. He was very much troubled with pain in his stomach and bowels, accompanied with dizziness in the head. The bowels would remain without a motion for a whole week unless a purgative was taken, in which instance an unusually large dose was required, and the pain attending its operation was represented as being dreadful The stool was hard, and of a light or brownish color. I might mention, also, that there was a sense of weight in the right hypochondriac region, sometimes amounting to downright pain. He remained in this condition for several years, when he was compelled to give up his avocation—school teaching—and turn his whole attention to the malady which was preying upon his system. All the cathartics were tried that seemed to promise any good in removing the costiveness, which was the most troublesome symptom in the case. He would use one article—for instance, Rhubarb—until the system lost its susceptibility of being acted upon by it, and then he would resort to another with the same result. Thus he continued up to the summer of 1849, when I commenced treating him with the inspissated ox bile. On the day that I gave him the first dose, he had had no motion of the bowels for four days. In the afternoon of that day he took two [10 grs.] pills, and repeated in four hours. The last dose was shortly afterwards followed by a full, soft and *painless* stool, to the great joy and satisfaction of the patient. He continued to take a pill, night and morning, until the most complete regularity of the bowels was established. The pain of the stomach and bowels entirely subsided, and his general health became much improved. He is almost of opinion that the *Fel Bovinum* is a specific, and prepares it himself, to use as occasion may require. This is only one of the many instances of the kind which could be mentioned, in which I have used this article with the most satisfactory results.

I shall mention another case, which is of a somewhat different class of patients, who have been signally benefitted by the remedy under consideration. Mrs. ——, aged about 22 years, light complexion and medium size. Was a resident, up to the summer of last year, of the State of Mississippi. She is slightly hysterical, and has been for years affected with costiveness to such an extent as to be under the necessity of taking physic every few days. Large doses were required, which, after a painful operation, left the bowels in their usual condition. She was somewhat chlorotic, and frequently suffered from what she called "fainting spells," which were very unpleasant indeed. After I had administered in this—as in the first mentioned—case, ordinary cathartics, with but temporary effect, I resorted to the inspissated ox bile. I evaporated the gall to the consistence of thick tar, and then brought it to the consistence of pill mass by the addition of the precipitated carbonate of iron, on 5 gr. pill of which was given three times per day, with the effect of establishing perfect regularity of the bowels. I had one pill taken daily for two months, and we had the satisfaction of seeing the complexion of the patient assume a more ruddy and healthy appearance, the fainting fits reduced in frequency, and the strength improved. It would be well to state that in this case, after about one month, the use of the inspissated gall and iron pills were suspended every other night, in order to admit of the administration of a small dose of taraxicum, for its alterative and tonic effect.

This article is of undoubted utility in cases of children laboring under diarrhea, where the stools are light colored, indicating a lack of the normal quantity of bilious secretion in the system. The well known power of bile, whether human or not, of preserving milk from coagulation has suggested the idea of using it in cases of infants upon whose stomachs the nurse's milk curdles, producing vomiting and irritability of that organ. This practice is said to be very satisfactory by those who have ample opportunity of testing it in such cases From the established fact, also, that a solution of gall, when poured even in very small quantities over hardened feces which have been voided from the body, reduces it to a soft, pulpy consistence, it has led the inductive medical practitioner to use it as an injection where the rectum is filled with impacted feces. I have used it with good effect in a marked case of this kind. We very plainly see that the mode of operation of this medicine is the same within as without the alimentary canal. Its contents are softened, thus enabling the natural peristaltic action to propel them onward. We can in this way account for their *painless*

operation, while those remedies which act merely by increasing the peristaltic motion produce such great pain. It may be urged that this medicine will prove insufficient from the fact that constipation is but a *symptom* of an unhealthy condition behind it; but whilst this may be true, it will be well to recollect that when constipation is once established as a secondary effect it has a baleful reaction upon the system, and we think that if this symptom be attended to, the first link in the chain of morbid action will often disappear of itself.—*Ohio Med. and Sur. Jour.*

